# Effects of concurrent training on the Chinese female elite triathletes

**DOI:** 10.1371/journal.pone.0329588

**Published:** 2025-08-13

**Authors:** Chenghao Liu, Zhenyu Zhang, Rui Mao, Li Xia, Yun Xie

**Affiliations:** 1 School of Exercise, Tianjin University of Sport, Tianjin, China; 2 School of Physical Education, Tianjin University of Sport, Tianjin, China; 3 School of Life Sciences, Tiangong University, Tianjin, China; eCampus University: Universita degli Studi eCampus, ITALY

## Abstract

This study investigated the effects of an 8-week concurrent strength-endurance training program on the performance of elite female triathletes in China. Twelve female national team members (age 18.41 ± 1.44 years) completed a phased concurrent training program, which included strength (three times per week) and endurance (three times per week) modules. TRIMP was used to monitor training load. The test indicators included 1RM strength, vertical jump (CMJ/SJ), swimming/running performance, and short- and medium-distance triathlon simulation races. The results showed that 1RM squat (+4.23% ± 2.99%, *P *< 0.001), relative squat strength (+3.96% ± 3.0%, *P* < 0.001), static jump (SJ, + 3.65% ± 1.42%, *P* < 0.001), 400-meter swimming (−0.85% ± 0.25%, *P* < 0.001), 2000-meter running (−0.47% ± 0.48%, *P* < 0.01), and short-distance triathlon performance (−3.63% ± 0.11%, *P* < 0.001) significantly improved. Maximum output power increased by 5.52 ± 3.06 W (*P* < 0.001), but VO_2max_ and medium-distance triathlon performance remained unchanged. Concurrent training effectively enhanced the lower limb explosive power and short-distance performance of elite female triathletes through neuromuscular adaptations, and the intervention effects were sustainable, supporting its integration into periodized training programs.

## Introduction

It is well-established that strength and endurance constitute fundamental physical attributes for athletes. In training practice, strength and endurance exercises exhibit distinct mechanistic effects on athletes due to their inherent physiological differences. Inappropriate design of training content, methodologies, and load parameters may diminish training efficacy or even induce conflicting adaptations. Over recent decades, sports scientists worldwide have extensively investigated concurrent training approaches [1–4]. While numerous studies demonstrate that concurrent training yields more favorable effects on strength and endurance compared to traditional segregated training modalities, conflicting evidence suggests potential inferior outcomes relative to isolated strength or endurance programs [5]. Consequently, mitigating incompatibility between strength development and endurance adaptations during concurrent training remains a critical challenge for coaches across disciplines.

Concurrent strength and endurance training has emerged as a focal research area in international sports science. In Europe and North America, over three decades of empirical exploration (since the 1980s) have established robust theoretical frameworks and practical methodologies widely applied in elite athletic training. Termed “Simultaneous Training” or “Concurrent Training,” this approach integrates resistance training (targeting strength, hypertrophy, and power) with endurance-enhancing protocols within the same training phase (Wilson, 2012) [6]. In contrast, research on concurrent training in China remains nascent, with limited peer-reviewed publications and a paucity of theoretical or empirical foundations.

Hickson (1980) [7], a pioneering American exercise physiologist, first investigated the compatibility of concurrent strength and aerobic Endurance training in 1980, concluding that such training impedes muscular strength adaptations while preserving aerobic gains. Notably, endurance-trained athletes exhibit superior compatibility with concurrent strength training compared to untrained individuals, demonstrating more pronounced performance enhancements (Hunter, 1987). Subsequent studies have focused on interference effects across key adaptation domains: power output [6], muscle hypertrophy [8–10], and maximal strength [2]. Consensus indicates that endurance training and resistance training induce divergent biological adaptations, with aerobic components attenuating resistance training-induced improvements in muscle mass, strength, and power, while minimally affecting aerobic capacity development [11].

International scholars posit that concurrent strength-endurance training synergistically enhances physiological capacity and movement economy, particularly in endurance-dominant disciplines like cycling and running [12,13]. Although limited literature directly examines concurrent training effects in triathlon, empirical evidence suggests that strategic programming—optimizing exercise selection, load parameters, movement velocity, timing, frequency, and session duration—can significantly improve triathletes’ performance [14]. Although the benefits of concurrent training have been confirmed by numerous studies, the majority of existing research predominantly focuses on male athletes, with female participants being underrepresented. This gender imbalance limits the applicability of findings across sexes and underscores the necessity for female-specific investigations. Extensive literature has highlighted the marked underrepresentation of female athletes in sports science research, particularly in studies related to athletic performance [15,16]. Therefore, while concurrent training has been shown to enhance athletic performance in males, its effectiveness in improving performance among female athletes remains unclear. Sex-specific differences are evident, particularly in morphological and physiological dimensions [17]. Morphologically, female athletes possess significantly lower skeletal muscle mass compared to their male counterparts, which contributes to reduced maximal strength capacity [18,19]. Moreover, females typically exhibit a higher proportion of type I muscle fibers (oxidative, fatigue-resistant) and greater capillary density, facilitating enhanced oxygen and nutrient exchange. In contrast, males have a higher proportion of type II fibers (glycolytic, power-oriented) [20–22]. Significant sex-related differences also exist in the respiratory system. Females have smaller lung volumes and narrower airways than males, resulting in greater work of breathing (Wb) and higher oxygen consumption by respiratory muscles [23,24]. Furthermore, women demonstrate superior diaphragmatic fatigue resistance and tend to recruit accessory respiratory muscles earlier during high-intensity exercise to offload the diaphragm [25,26]. From a physiological perspective, females exhibit greater fat oxidation capacity and reduced glycolytic activity, leading to lower ATP depletion post-exercise and higher mitochondrial respiration rates [27]. Additionally, females display slower skeletal muscle calcium kinetics and lower sarcoplasmic reticulum Ca^2+^ -ATPase activity, resulting in delayed metabolic perturbations and later onset of fatigue at equivalent exercise intensities [28]. Hormonal factors, such as estrogen, also influence neuromuscular excitability in females [29]. Collectively, these multidimensional physiological differences between sexes may result in sex-specific responses to training load, adaptive mechanisms, and performance outcomes under concurrent training. Therefore, independent empirical studies focusing on female athletes are urgently required to validate performance adaptations and response trajectories under concurrent training paradigms.

Despite advancements, critical research gaps persist regarding triathlon-specific concurrent training: limited causal evidence, small sample sizes, inadequate control for individual variability, and insufficient multidimensional outcome assessments. To address these limitations, we propose an experimental intervention study to (1) quantify direct effects of concurrent training on elite female triathletes’ performance and (2) evaluate long-term impacts on holistic performance and health outcomes. Resolving these questions will advance evidence-based training optimization, injury prevention, and scientific training paradigms in triathlon, yielding substantial theoretical and practical implications. (3) To enhance the performance of the Chinese women’s triathlon team in mixed relay events and ultimately improve China’s overall triathlon results in World Championships and the Olympic Games.

## Materials and methods

### Participants

Twelve elite female triathletes were recruited for this study between October 7 and October 14, 2024 (Table 1). All participants met the following inclusion criteria: (1) aged 15 years or older; (2) no prior exposure to concurrent training methodologies; and (3) free from exercise-related medical contraindications or musculoskeletal injuries in the three months preceding the study. (4) According to the Participant Classification Framework proposed by McKay et al. [30], the athletes recruited for this study can be classified as Tier 3 (Sub-Elite/National-Level). All participants were members of the Chinese national triathlon team training pool and regularly competed in high-level domestic competitions sanctioned by the General Administration of Sport of China.

**Table 1:** Overview of basic information of participants.

Indicators	Mean ± SD
Age	18.41 ± 1.44
Height (m)	1.64 ± 0.04
Weight (kg)	52.68 ± 4.55

Informed consent was obtained from all participants prior to their inclusion in the study. For those under the age of 18, written informed consent was also obtained from their legal guardians. The research protocol strictly adhered to the principles outlined in the Declaration of Helsinki (2013 revision), ensuring that participants were fully informed of the study purpose, procedures, potential risks, and benefits, and that their rights, safety, and well-being were protected throughout the study.

This study involved no intervention, deception, or collection of sensitive personal data. All procedures were observational and non-invasive. According to the ethical guidelines of the Ethics Committee of Tianjin University of Sport, the research was classified as low risk and therefore exempt from formal ethics review (S1 and S2 Files).

### Procedure

An 8-week intervention training program was administered to all athletes. Testing was conducted at the beginning and end of the training period, with each assessment phase spanning seven days (Table 2). The testing protocol was structured as follows: Day 1: Height and body weight, countermovement jump (CMJ), and static squat jump (SJ). Day 2: 400m swimming (morning) and 1RM in squat (afternoon). Day 3: 5000m running, 2000m Cycling (morning) and anaerobic power (afternoon). Day 4: 1500m swimming (morning) and 1RM in bench press (afternoon). Day 5: 2000m running, 2000m Cycling (morning) and VO_2max_ (afternoon). Day 6: Sprint-distance triathlon test (SDT, 0.3 km swim + 6.6 km cycling + 2 km run). Day 7: Half-distance triathlon test (HDT, 0.75 km swim + 20 km cycling + 5 km run). Within 3 minutes after completing the test, the subjects were asked to report their sRPE to reflect their self-assessment of the training load.

**Table 2:** Testing schedule.

Days	Morning	Afternoon
1	Height; Weight; CMJ^; SJ^	
2	400m Swimming	Squat^
3	5000m Running and 2000m Cycling	Anaerobic Power
4	1500m Swimming	Bench Press^
5	2000m Running and 10000m Cycling	VO_2max_ Test
6		Sprint-Distance Triathlon
7		Half-Distance Triathlon

^: Test three times and record the best result.

After the intervention experiment, an 8-week follow-up period was conducted. At the end of the follow-up, physiological indicators that showed significant changes during the intervention were re-tested to explore whether regression would occur after returning to the traditional training mode.

Body composition indicators, including body fat percentage and skinfold thickness, were measured using a body composition analyzer (InBody 720), an anthropometric measuring tape, and Harpenden skinfold calipers. Height and circumference were measured to the nearest 1 cm, and skinfold thickness to the nearest 1 mm. Participants fasted for 8 hours prior to testing.

Since the participants were elite athletes, strength was assessed using the 1RM direct testing method. A full-frame squat rack was used for the deep squat, and a flat bench press rack was used for the bench press. A standard women’s 15 kg barbell and 45 cm Olympic weightlifting plates were used as testing equipment. Based on each athlete’s previously measured 1RM, an initial load was selected for the first lift. If the attempt was successful, the weight was increased in 2.5 kg increments until the athlete failed three consecutive attempts. The last successfully lifted weight was recorded as the athlete’s 1RM. If the athlete failed all three initial attempts, the load was reduced by 10 kg and then increased in 2.5 kg steps until a successful lift was achieved.

Explosive strength was assessed using a force platform test, applied identically for both the countermovement jump (CMJ) and squat jump (SJ). Athletes stood on the force platform to prepare, and upon hearing the start command, performed a maximal effort jump. The platform recorded flight time and used it to calculate jump height. Simultaneously, video footage was captured using an iPhone at 240 FPS to aid in confirmation. To standardize data recording and analysis, CMJ and SJ heights were rounded to the nearest 0.5 cm. Each athlete completed three trials for both CMJ and SJ, and the best result was recorded as the final score. (h: jump height; g = 9.81 m/s^2^; t: flight time)


$$\begin{array}{c} h=\frac{1}{8}gt^{2\;}\; \end{array}$$ (1)

The 2000-meter and 5000-meter running tests were conducted on a standard 400-meter track, with all athletes starting in the same group. Results were recorded in seconds, rounded to one decimal place. The 400-meter and 1500-meter swimming tests were conducted in a standard 50-meter pool, with four athletes starting per group. Participants completed 4 and 15 laps respectively, and the results were also recorded in seconds to one decimal place. The 2000-meter and 10,000-meter cycling tests were conducted on-road at the athletes’ regular training venue, with GPS used to determine the test distance. All athletes started together in the same group. The maximal oxygen uptake test was performed using a Cortex Metalyz spirometry tester and a running table, using the classic Bruce protocol, with the following end conditions: 1) subject oxygen uptake remained essentially unchanged, or even decreased slightly, for sustained increases in incline speed; 2) respiratory quotient ≥ 1.10; and 3) the highest heart rate recorded during the workout was achieved.

Anaerobic capacity was assessed using a MONARK 894E power bike following the standard Wingate test protocol.

#### Training load control.

Some scholars use Training Impulse (TRIMP) as a comprehensive indicator of training and competition load. Since TRIMP is calculated based on exercise intensity (average exercise heart rate) and duration, it can be used to measure the internal and external loads of intermittent exercise competitions. Andre S. Martorelli and other scholars analyzed [31] the impact of three different resistance training programs on internal load in 2020 and the correlation between internal and external loads, finding significant correlations (r = 0.35–0.80; *P*≤ 0.05) between external and internal stress indicators for all programs. Therefore, TRIMP can be selected as an effective indicator for measuring the internal and external loads of strength training. Based on these theories, we have chosen TRIMP alone as a training monitoring method rather than combining it with other methods. We believe this is sufficient to measure the load combination of our training [32].

### Training plan

#### Strength training.

**Phase 1 (Weeks 1–3: Strength Adaptation Period):** Comprehensive Full-Body Strength Training: Athletes performed foundational strength exercises (back squats, bench presses, deadlifts) with 3–4 sets of 8–10 repetitions at 60–70% of 1RM (adjusted per individual capacity). Emphasis was placed on proper form and neuromuscular activation, with moderate intensity (2–3 minutes rest between sets), to establish a baseline for subsequent training.

Core Stability Training: Exercises included front planks, side planks, and supine leg raises, performed for 45–60 seconds per set (3–4 sets total). Rest intervals of 1–2 minutes between sets maintained moderate intensity, targeting core muscle activation and postural stability.

Upper-Body Auxiliary Strength Training: Drills such as dumbbell shoulder presses and pull-ups (or assisted pull-ups) were conducted with 2–3 sets of 8–10 repetitions at light loads. Rest intervals of 2 minutes prioritized movement control and muscular endurance to promote balanced strength development.

**Phase 2 (Weeks 4–6: Foundational Strength Period):** Power Development Training: Explosive elements were integrated into foundational lifts (e.g., ballistic squat jumps, bench press throws) with 3–4 sets of 6–8 repetitions at 70–80% of 1RM (1RM refers to the maximum load that an athlete can lift once with proper form during a squat jump). Extended rest periods (3–4 minutes) supported high-intensity efforts focused on rate of force development and elastic muscle contraction.

Advanced Core Strength Training: Complexity was increased through unstable-surface exercises (e.g., Swiss ball planks, supine knee tucks), sustained for 60–90 seconds per set (3–4 sets). Reduced rest intervals (1–2 minutes) enhanced core strength and stability under sport-specific demands.

Sport-Specific Strength Training: Triathlon-specific drills included: (1) Swim: Elastic band resistance training mimicking stroke mechanics (2) Cycling: Fixed-gear machine exercises replicating cycling postures (3) Run: Hill sprint repeats. Protocols involved 3–4 sets of 10–12 repetitions, with load/velocity tailored to discipline-specific biomechanics.

**Phase 3 (Weeks 6–8: Rapid Strength Period):** Maximal Strength Consolidation: Foundational lifts (squats, bench presses, deadlifts) were maintained at 3–4 sets of 4–6 repetitions (80–90% of 1RM). Prolonged rest intervals (3–4 minutes) maximized neuromuscular adaptation for strength retention and power endurance.

Integrated Strength-Technique Training: Sport-specific overload strategies included: (1) Swim: Weighted swim drills using buoyancy vests or resistance parachutes (2) Cycling: High-intensity interval sessions simulating race-specific climbs/sprints (3) Run: Technique-focused interval sprints. Emphasis was placed on translating strength gains into technical proficiency.

Functional Movement Training: Multidirectional agility drills (e.g., ladder footwork, balance beam walks, plyometric jumps) were performed for 3–4 sets of 10–15 repetitions. Moderate-intensity protocols targeted coordination, flexibility, and reactive capacity to enhance race-situation adaptability (Table 3).

**Table 3:** Eight-week strength training program.

Training Phase	Exercise	Load	Sets×Reps	Rest Interval
Phase 1 (Weeks 1–3)	Full-Body Strength Training (Squat, Bench Press, Deadlift)	60–70% 1RM	3–4 × 8–10	2–3 min
	Core Stability Training (Plank Variations, Leg Raises)	Bodyweight	3–4× 45–60sec	1–2 min
	Upper Body Assistance (Dumbbell Shoulder Press, Pull-Ups)	Light load/ Bodyweight	2–3 × 8–10	2 min
	Bodyweight Lunges (Assistance Exercise)	Bodyweight	3 × 12 (each leg)	1–1.5 min
	Weighted Calf Raises (Gastrocnemius Focus)	Bodyweight/ 10 kg Dumbbell	3 × 15	1 min
Phase 2 (Weeks 4–6)	Power Training (Jump Squats, Med Ball Chest Throws)	70–80% 1RM	3–4 × 6–8	3–4 min
	Advanced Core (Unstable Core Drills)	Bodyweight/ Swiss Ball	3–4× 60–90sec	1–2 min
	Swim-Specific Strength (Resistance Band Pulls)	Medium-Resistance Band	3–4 × 10–12	1.5 min
	Bike-Specific Power (High RPM Fixed Gear Intervals)	Moderate Load	3–4 × 12	2 min
	Run-Specific Strength (Uphill Sprinting)	Bodyweight	3–4× 30–40m	2.5–3 min
	Seated Row (Postural & Swim Mechanics)	Moderate Weight	3 × 10	1.5 min
Phase 3 (Weeks 6–8)	Maximal Strength Maintenance (Squat, Bench Press, Deadlift)	80–90% 1RM	3–4 × 4–6	3–4 min
	Integrated Sport Simulation (Resistance Swim/Bike/Run)	High Resistance Tools	3–4× 50%SDT	2–3 min
	Functional Movement (Agility, Balance, Plyos)	Bodyweight or Light Resistance	3–4× 10–15	1.5–2 min
	Single-Leg Squats (Balance & Core)	Bodyweight/ Assistance Band	3 × 8 (each leg)	1.5 min
	Resisted Shuttle Runs (Tethered or Band)	Moderate Resistance	4 × 20m × 2	2 min

#### Endurance training.

**Aerobic endurance training:** Long-Duration Endurance Training: Athletes performed 2–3 weekly sessions of extended-duration swims (2,000–4,000m), cycling (90–150 minutes), and runs (10–15 km) at low-to-moderate intensity (60–70% of maximum heart rate [HR_max_ = 220 − age]). These sessions prioritized aerobic capacity development and cardiorespiratory efficiency through sustained energy system engagement.

Interval Training: (1) Running: 8–10 sets of 400m high-intensity runs (80–90% of maximum speed, Maximal speed refers to the best average speed achieved over this subject, same below.) alternated with 200m active recovery jogs, with 1–2 minutes rest between sets. (2) Swimming: 6–8 sets of 100m maximal-effort sprints followed by 50m easy-paced recovery swims. (3) Cycling: 10–12 sets of 30-second all-out sprints interspersed with 1–2 minutes of low-intensity pedaling. Conducted 1–2 times weekly, this protocol aimed to elevate anaerobic threshold and enhance hybrid aerobic-anaerobic metabolic adaptability.

**Sport-Specific Endurance Training:** Competition Simulation Drills: Athletes completed 1–2 weekly full-course triathlon simulations (swim-cycle-run sequence) with progressive overload in intensity to acclimatize to race-pace demands and energy expenditure patterns.

Discipline-Specific Intensity Training: (1) Swim: Repeated maximal-effort sprints to improve race-start and overtaking capabilities. (2) Cycling: High-resistance gradient climbing drills mimicking elevation challenges. (3) Run: Race-pace interval sprints targeting finish-line surge capacity. These sessions focused on optimizing power output and endurance resilience during critical competition phases.

**Fig 1:**
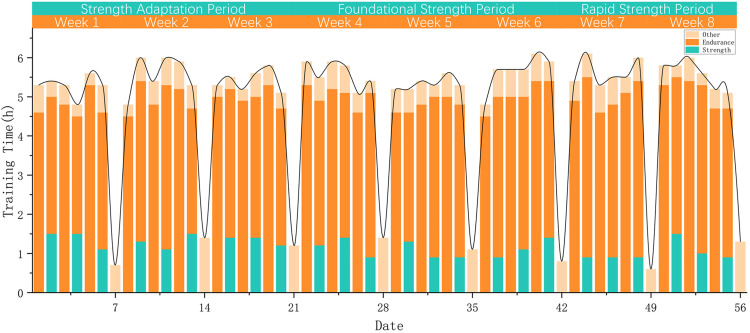
Actual daily training time. The figure shows the total duration of daily training sessions during the intervention period. Endurance sessions occurred on Mondays, Wednesdays, and Fridays, while combined strength-endurance sessions were conducted on Tuesdays, Thursdays, and Saturdays. Sundays were used for active recovery. Training time was maintained at approximately 5.5 hours per day.

During the experimental design phase, we anticipated variations in training duration across different training modalities; however, the total training time across the three phases was expected to remain relatively consistent. Specifically, endurance training was prioritized on Mondays, Wednesdays, and Fridays, while strength and endurance training were combined on Tuesdays, Thursdays, and Saturdays. Sundays were designated as recovery days, primarily involving stretching and other low-intensity activities. To prevent overtraining, the total daily training duration was planned to be limited to approximately 5.5 hours. Analysis of actual training data showed that the training load closely aligned with the planned schedule, indicating that the program was well implemented in practice (Figs 1, S1 and S3 Table).

### Statistical analysis

Data organization and analytical procedures were conducted using IBM SPSS Statistics 25 and Microsoft Excel. Because of the small sample size of the study (N = 12), the normality of the distribution was verified by the Shapiro-Wilk (S-W) test. For datasets adhering to normality assumptions, paired-sample *t*-tests were employed to assess pre-post intervention differences. Statistical significance thresholds were defined as *P* < 0.05, *P* < 0.01, and *P* < 0.001. The effects are calculated using Hedges’ g Modified Cohen’s d for small samples in study.

## Results

During the 8-week intervention period, the total TRIMP (Training Impulse) was 1190.5 ± 93.15. Strength training contributed 9.10% (108.33 ± 21.60) to the total load, while endurance training constituted the majority: swimming (205.17 ± 21.84, 16.39%), cycling (500.75 ± 56.83, 40.38%; the largest endurance component), and running (372.50 ± 43.03, 31.29%). Supplemental training modalities (flexibility, agility, and small-muscle-group exercises) accounted for the smallest proportion (33.75 ± 18.1, 2.83%).

**Fig 2:**
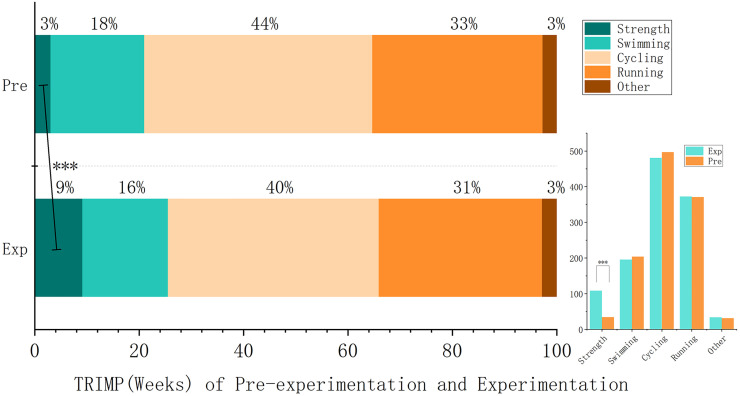
Comparison of weekly average TRIMP between pre-experiment and experiment. This figure compares the average Training Impulse (TRIMP) values per week between the pre-intervention phase and the intervention phase. While total TRIMP remained stable, strength training TRIMP increased significantly, indicating an intentional redistribution of training load.

After the Shapiro-Wilk test, all data conformed to a normal distribution (*p* > 0.05). Compared to pre-intervention levels, total TRIMP showed no statistically significant change (4.19% ± 8.25%, *P* > 0.05), with considerable inter-individual variability. Among endurance components, swimming (−4.88% ± 9.09%, *P* > 0.05), cycling (−4.03% ± 9.13%, *P* > 0.05), and running (0.22% ± 8.52%, *P* > 0.05) exhibited non-significant fluctuations. In contrast, strength training demonstrated a highly significant increase (67.18% ± 7.62%, *P* < 0.001), rising from a baseline contribution of 3.01% to 9.10% (S1 Table, Table 4, Figs 2 and S2).

**Table 4:** Overview of TRIMP in 8 weeks intervention training and 8 weeks pre-intervention training.

Indicators		Pre-Intervention N = 8		Intervention Phase N = 8		Change rate (%)	Hedges’ g
Level I	Level II	TRIMP	Rate (%)	TRIMP	Rate (%)		
Total		1136.25 ± 85.81	100	1190.5 ± 93.15	100	4.19 ± 8.25	0.49
Strength		34.17 ± 2.41	3.01	108.33 ± 21.6***	9.10	67.18 ± 7.62	3.08
Endurance	Swimming	203.75 ± 20.74	17.93	195.17 ± 21.84	16.39	−4.88 ± 9.09	−0.47
	cycling	496.75 ± 43.85	43.72	480.75 ± 56.83	40.38	−4.03 ± 9.13	−0.34
	Running	370.67 ± 45.84	32.62	372.5 ± 43.03	31.29	0.22 ± 8.52	0.05
Other		30.92 ± 14.8	2.72	33.75 ± 18.1	2.83	2.11 ± 21.02	0.31

*P < 0.05, **P < 0.01, ***P < 0.001 (The Same Below) (Duration of Each Phase = 8 Weeks).

**Fig 3:**
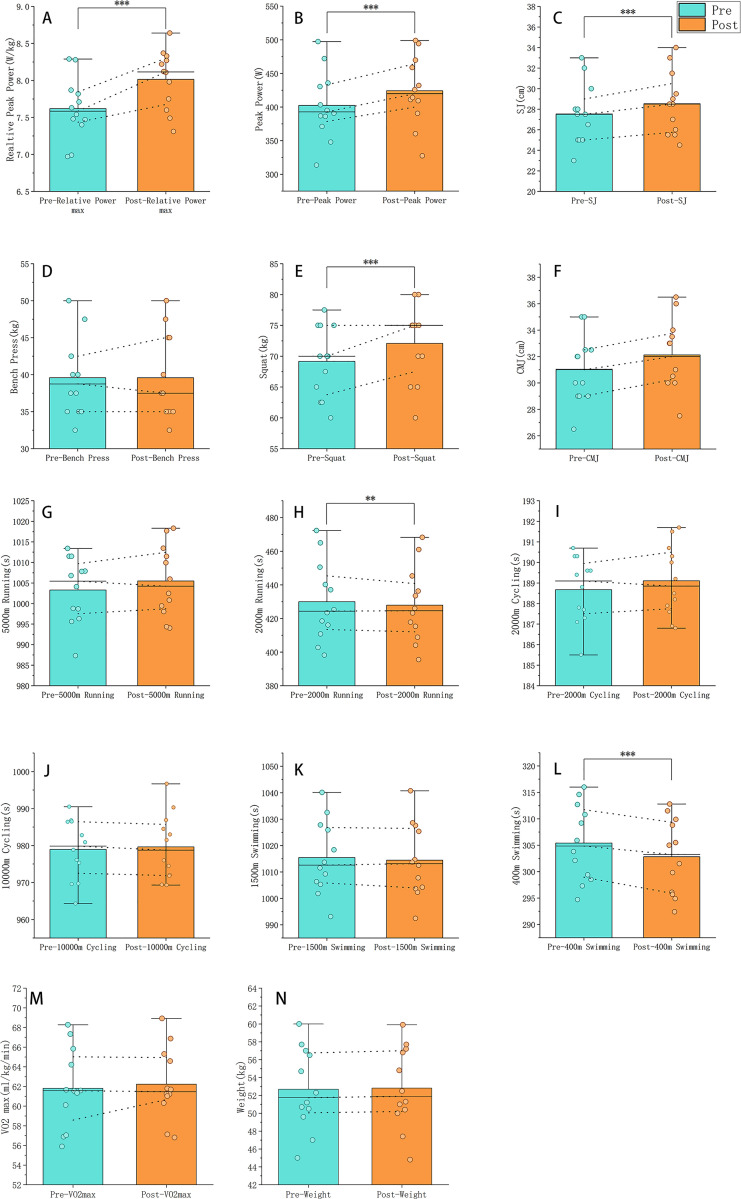
Continued.

The 12 female triathletes exhibited minimal body weight changes (0.99% ± 0.26, *P* > 0.05). Significant improvements were observed in squat strength (4.23% ± 2.99%, *P* < 0.001) and relative squat strength (3.96% ± 3.0%, *P* < 0.001). Bench press performance showed no meaningful changes (absolute: −0.1% ± 3.72%, *P* > 0.05; relative: −0.34% ± 3.98%, *P* > 0.05). Countermovement jump (CMJ) demonstrated a non-significant increase (3.64% ± 5.71%, *P* = 0.064), whereas static squat jump (SJ) improved markedly (3.65% ± 1.42%, *P* < 0.001) 400m swim time decreased significantly (0.85% ± 0.25%, *P* < 0.001). 2000m run performance improved substantially (0.47% ± 0.48%, *P* < 0.01). Sprint-distance triathlon completion time showed pronounced enhancement (3.63% ± 0.11%, *P* < 0.001). 1500m swim (0.1% ± 0.21%, *P* > 0.05) and 5000m run (−0.22% ± 0.43%, *P* > 0.05) exhibited no statistically significant changes. Half-distance triathlon performance improved marginally (0.28% ± 0.19%, *P* > 0.05) without reaching significance (Table 5, S2 Table, Figs 3, S3 and S4).

**Table 5:** Overview of fitness and specialized results.

Indicators	Pre	Post	Change Rate (%)	Hedges’ g
Weight (kg)	52.68 ± 4.55	52.82 ± 4.53	0.99 ± 0.26	−0.50
400m Swimming(s)	305.42 ± 7.21	302.82 ± 7.08***	0.85 ± 0.25	3.12
1500m Swimming(s)	1015.47 ± 13.81	1014.46 ± 13.77	0.1 ± 0.21	0.43
2000m Running(s)	430.01 ± 23.56	427.93 ± 22.16**	0.47 ± 0.48	0.91
5000m Running(s)	1003.31 ± 7.96	1005.5 ± 8.59	−0.22 ± 0.43	−0.47
2000m Cycling(s)	188.68 ± 1.59	189.11 ± 1.71	0.23 ± 0.45	0.48
10000m Cycling(s)	978.97 ± 8.18	979.66 ± 8.88	−0.07 ± 0.59	0.11
Squat 1RM(kg)	69.17 ± 5.77	72.08 ± 6.2***	4.23 ± 2.99	−1.31
Relative Squat 1RM (%)	131.5 ± 7.43	136.8 ± 10.23***	3.96 ± 3.0	−1.21
Bench Press 1RM (kg)	39.58 ± 5.31	39.58 ± 5.82	−0.1 ± 3.72	0.00
Relative Bench Press 1RM (%)	75.38 ± 9.5	75.14 ± 10.21	−0.34 ± 3.98	0.07
CMJ (cm)	31.04 ± 2.57	32.12 ± 2.66	3.64 ± 5.71	−0.56
SJ (cm)	27.54 ± 2.97	28.54 ± 3.06***	3.65 ± 1.42	−2.54
HDT (H:Min.S)	01:00:39 ± 00:00.54	01:00:28 ± 00:00.56	0.28 ± 0.19	0.43
SDT (H:Min.S)	00:24:46 ± 00:00.43	00:23:54 ± 00:00.41***	3.63 ± 0.11	5.76

Physiologically and psychologically, VO_2max_ exhibited negligible change (0.76% ± 2.45%, *P* > 0.05). However, both absolute (5.52 ± 3.06 W) and relative (5.24 ± 2.82 W/kg) peak power (Wingate test) output increased significantly (*P* < 0.001), though substantial inter-individual variability in response magnitude was observed. However, based on the sRPE, no significant changes were observed between the pre- and post-tests, with values remaining consistently between 17 and 18 (Table 6 and Fig 3).

**Table 6:** Overview of physiological and psychology indicators.

Indicators	Pre	Post	Change Rate (%)	Hedges’ g
VO_₂max_ (ml/kg/min)	61.81 ± 4.04	62.24 ± 3.62	0.76 ± 2.45	−0.29
Peak Power (W)	402.48 ± 50.72	424.36 ± 51.25***	5.52 ± 3.06	−1.68
Relative Peak Power (W/kg)	7.62 ± 0.42	8.02 ± 0.40***	5.24 ± 2.82	−1.76
sRPE	17.5 ± 0.65	17.83 ± 0.9	2.04 ± 6.31	−0.27

**Fig 4:**
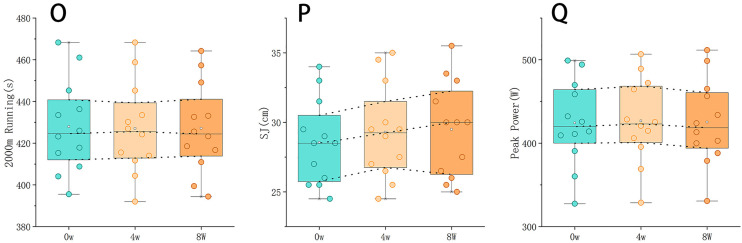
Indicators change at 0, 4 and 8 weeks of follow-up period (mean±sd) (O:2000m running; P:Peak Power; Q:SJ). (O) 2000-meter running time (s) measured at 0, 4, and 8 weeks post-intervention, showing no significant performance decline. (P) SJ height (cm) across the same time points, indicating stable lower-body explosive strength. (Q) Absolute peak power (W) from Wingate test results, demonstrating sustained anaerobic performance levels. Each data point represents the group mean ± SD. No statistically significant regression was observed in any variable over the 8-week follow-up period, suggesting that performance adaptations achieved through concurrent training were maintained even after returning to a traditional training regimen.

After the intervention experiment, the researchers selected SJ, Peak Power, and 2000m running as the re-test indicators. Testing was conducted every four weeks during the follow-up period, and each test was performed once to determine the result. The experimental results showed no significant changes in SJ, Peak Power, or 2000m running (*P* > 0.05) (S4 Table, Figs 4 and S5).

## Discussion

### Differential performance

The 8-week intervention program allocated 9.10% of the total training load (TRIMP) to strength training. Results revealed significant improvements in squat performance (4.23% ± 2.99%, *P* < 0.001) and relative squat strength (3.96% ± 3.0%, *P* < 0.001), underscoring the program’s efficacy in enhancing lower-body power. In contrast, bench press metrics—both absolute (−0.1% ± 3.72%) and relative (−0.34% ± 3.98%)—remained statistically unchanged (*P* > 0.05), suggesting limited upper-body adaptation. Jump performance exhibited divergent outcomes: static squat jump (SJ) improved markedly (3.65% ± 1.42%, *P* < 0.001), while countermovement jump (CMJ) showed a non-significant upward trend (3.64% ± 5.71%, *P* = 0.064). These findings indicate partial success in power development, with room for optimization in neuromuscular coordination strategies.

Some variables demonstrated exaggerated effect sizes, such as 400 m swimming (Hedges’ g = 3.12) and SDT (Hedges’ g = 5.76). After repeated data verification, the authors believe these large values may be attributed to the high homogeneity of the sample—reflected by low standard deviations—and the fact that the intervention was specifically designed to improve SDT performance, making a significant post-intervention improvement plausible. However, even with the correction provided by Hedges’ g, caution is warranted when interpreting these unusually large values. The small sample size (N = 12), potential measurement artifacts, and low variability may have contributed to the inflation of effect sizes. Future studies involving larger and more heterogeneous samples are recommended to validate these results.

Notably, our athletes exhibited only a 4.23% improvement in squat performance, which contrasts with the findings of Støren et al. (2008) [33] and Sunde et al. (2010) [34], who reported approximately 20% strength gains in runners and cyclists following 8–10 weeks of training. It is important to emphasize that our training design differed substantially from that of Støren et al. (2008) [33] and Sunde et al. (2010) [34]. Their protocols focused primarily on isolated, high-load resistance training, whereas our intervention modified the original training framework by increasing the proportion of strength training and reducing endurance volume, which may have influenced the extent of strength gains. Additionally, our participants were national-level elite athletes, whereas the subjects in the aforementioned studies were trained or moderately trained individuals, which may have contributed to differences in strength development outcomes. Specifically, certain performance capacities in high-level athletes may approach their genetic potential. For instance, Millet et al. [35] reported a 6% improvement in running economy following concurrent training in 15 well-trained triathletes, while Vikmoen et al. [36] observed a 4.9% improvement in national-level female triathletes. These findings further suggest that the efficacy of concurrent training may be modulated by the athlete’s training background and experience level.

The observed strength adaptations likely stem from the program’s phased periodization—structured into strength adaptation, foundational strength, and rapid strength phases. During the foundational phase, progressive overload in squat training (volume and intensity) aligned with hypertrophy-strength adaptation principles, driving lower-body gains [37]. However, the lack of upper-body progress may relate to suboptimal exercise selection (e.g., a lower training load of the bench press), load prescription, or insufficient training frequency. Additionally, upper-body muscle groups may exhibit slower adaptive responses compared to lower-body counterparts, necessitating longer intervention periods or alternative programming [38].

The disparity between SJ and CMJ outcomes highlights biomechanical and neuromuscular complexities [39]. SJ primarily reflects instantaneous concentric power [40], whereas CMJ incorporates stretch-shortening cycle efficiency and intermuscular coordination [41]. The non-significant CMJ improvement suggests that technical refinement and proprioceptive training—targeting movement sequencing and elastic energy utilization—could further enhance explosive power translation.

### Physiological mechanism

The experimental results show that after 8 weeks of concurrent strength and endurance training intervention, the athletes’ squat strength and static squat jump significantly improved. The physiological mechanism can be explained by the AMPK-mTOR signaling interference hypothesis. AMPK (adenosine monophosphate-activated protein kinase), as a key inhibitor of the AKT-mTOR pathway [2,42–44], plays a crucial role in regulating muscle hypertrophy [45,46]. Continuous endurance exercise can induce AMPK phosphorylation, and the degree of its activation is dose-dependently related to the inhibitory effect on muscle hypertrophy: that is, the higher the intensity and volume of endurance training, the more significantly AMPK activity is enhanced [2,45], thereby inhibiting muscle protein synthesis mediated by the mTORC1 complex [45,46]. In this study, all the subjects were national-level athletes. By maintaining the total training load unchanged, increasing the duration of strength training and moderately reducing the proportion of endurance training, it was observed that the athletes’ 1RM squat, relative squat strength and static vertical jump (SJ) levels significantly improved. This phenomenon may be related to the upregulation of the mTORC1 signaling pathway due to the increase in strength training load, while the activation degree of AMPK decreases, thereby weakening its inhibitory effect on muscle hypertrophy and ultimately promoting neuromuscular adaptation and strength gain.

The performance in middle-distance events (such as 2000-meter run) not only depends on maximal oxygen uptake (VO_₂max_), but is also closely related to aerobic endurance and exercise economy [47]. This experiment found that after 8 weeks of intervention, the 400-meter swimming, 2000-meter run, and short-distance triathlon performances significantly improved, consistent with the recent meta-analysis results [48]: the concurrent training of the medium-term plan can significantly enhance the competitive performance in multi-event middle and long distances (>75 seconds) by optimizing the synergistic effect of explosive power and endurance adaptation. Specifically, the explosive power module in concurrent training enhances the elasticity of the muscle-tendon complex and improves the efficiency of the stretch-shortening cycle (SSC), thereby reducing energy dissipation during movement. This improvement in elasticity enables athletes to exert force more efficiently in periodic movements (such as running cadence, swimming arm strokes), delaying local muscle fatigue.

In addition, middle-distance events rely on both the aerobic oxidation system (maintaining steady-state output) and the anaerobic glycolysis system (responding to intermittent sprints). This program enhances VO_₂max_ and lactate threshold through long-duration aerobic training, supplemented by high-intensity interval training (such as 400-meter swimming sprints) to strengthen anaerobic metabolic capacity. The combination of the two enables athletes to efficiently switch energy supply modes during competition, significantly increasing the fatigue tolerance threshold. This mechanism may explain the significant improvement in 2000-meter run performance, while the improvement in long-distance events such as 5000-meter run is relatively limited due to the difference in the dominance of energy supply systems.

### Long-term effect

During the 8-week follow-up period, specific performance (2000m Running), explosive power (SJ), and physiological indicators (Peak Power) were maintained at post-intervention levels. This demonstrates that the effects induced by Concurrent Training can be retained even without ongoing training, suggesting that this method may have a long-term impact on physical adaptation. This implies that Concurrent Training can be incorporated as a block into the training programs of elite triathletes, serving as a “strength enhancement block” or a “middle-to-short distance enhancement block”.

Since the mixed relay event (300m + 6.8 km + 2 km) places high demands on an athlete’s middle-to-short distance ability, this design can help athletes better adapt to the mixed relay event. Following the 8-week concurrent training intervention, the training load distribution was reverted to the traditional balance of strength and endurance modalities. The results indicated that the positive effects of concurrent training on athletic performance did not significantly diminish. This suggests that once the adaptations induced by concurrent training are established, maintaining them may require only a minimal dose of continued strength training. This finding aligns with Rønnestad et al. (2010) [49], who observed that trained cyclists were able to maintain—or even slightly improve—power output and cycling economy by performing just one high-load strength training session per week during the competitive season.

However, the re-test did not assess all indicators. Therefore, over time, certain indicators may gradually decline. Future research could further explore how to optimize the maintenance of effects through the strategic arrangement of recovery and training cycles, providing more scientifically-based training recommendations for athletes.

### Hormonal fluctuations across the menstrual cycle and training adaptations

The phenomenon of menstrual cycle synchronization among females living in close proximity, such as in shared dormitories or training camps, has been substantiated by numerous studies [50]. In this study, the 12 elite athletes resided together throughout the preparation period, resulting in a high degree of cycle synchronization; consequently, only two participants were in the menstrual phase during the testing period. Although most athletes were not directly affected by menstruation during the testing period, the potential impact of the menstrual cycle on athletic performance should not be overlooked. Firstly, estrogen levels rise significantly during the follicular phase, particularly around ovulation, creating a hormonal environment that may enhance muscular adaptations to strength training. This is supported by the findings of Sung et al. [51], who demonstrated that in eumenorrheic women not using oral contraceptives, concentrating high-intensity training in the follicular phase led to significantly greater gains in muscle strength and cross-sectional area. Empirical research by Kissow et al. [52] further confirmed that high-intensity resistance training during the follicular phase, compared to the luteal phase, resulted in approximately 8% greater strength gains and a 5% increase in muscle cross-sectional area. Secondly, specific attention should be paid to the potential interference of the menstrual phase on test outcomes. A meta-analysis by McNulty et al. [53] indicated that athletic performance may be negatively affected when testing occurs in the early follicular phase (i.e., menstrual onset) compared to the late follicular phase, with a small but statistically significant decline observed. Moreover, a recent systematic review suggested that early menstruation may be associated with increased fatigue and a heightened risk of muscle damage [54].

Therefore, both the testing and follow-up phases were scheduled at 4- and 8-week intervals, with the aim of aligning pre- and post-test assessments within the same physiological menstrual cycle. This approach was intended to minimize potential bias resulting from testing during different menstrual phases. In practice, this scheduling was effective as intended; thus, even if some athletes were in the menstrual phase during testing, their results were still reported as valid samples. In summary, the various phases of the menstrual cycle may influence training adaptations and athletic performance through hormonal fluctuations. Therefore, it is recommended that coaches incorporate menstrual cycle tracking and individualized adjustments into training programs. Scientific periodization tailored to each athlete’s menstrual rhythm may help maximize muscular adaptations and ensure performance stability.

### Research limitations and prospects

This study involved only 12 elite female triathletes as participants. As a small-sample experiment, its generalizability may be limited, making it difficult to extrapolate the findings to a broader population directly. Additionally, considerable individual differences were observed among athletes in certain metrics (Such as bench press). Although standardized interventions were applied, variations in individual adaptability and response could affect the accuracy of the results. Therefore, future research should account for these individual differences and incorporate a larger sample size to enhance the study’s validity. Considering the athletes’ competition schedules, training cycles, and other practical factors, as researchers, we do not possess the authority to alter the coaches’ training plans,conducting a randomized controlled trial (RCT) might not have received approval from sports governing bodies. To ensure the timeliness and applicability of the findings, we opted for a single-arm study design while striving to control potential confounding factors. We recommend that future research, where feasible, incorporate RCTs to further validate these findings and control for potential biases.

In selecting experimental indicators, we prioritized the protection of participants, as all subjects were under the jurisdiction of the Chinese sports administration. To respect athletes’ privacy and well-being, invasive testing methods such as blood and urine tests were deliberately avoided. This decision was made out of a strong commitment to athlete privacy and in accordance with ethical committee regulations regarding study design. Although non-invasive measures such as heart rate (TRIMP), maximal oxygen uptake (VO_₂max_), and perceived exertion were used to monitor physiological status, these indicators, while reflective of physiological and psychological load to some extent, may not fully reveal deeper physiological mechanisms such as hormonal fluctuations and immune function. Therefore, the results primarily demonstrate the positive effects of concurrent training in practical training settings but do not allow for a detailed biochemical exploration of the underlying physiological mechanisms.

Although the non-invasive indicators used in this study provided valuable physiological data, we acknowledge that the absence of more precise assessments, such as blood and urine tests, may have left certain physiological mechanisms unexplored. Therefore, we recommend that future research incorporate emerging non-invasive technologies or, when privacy and health considerations permit, gradually adopt a more diverse range of assessment methods.

## Conclusion

The 8-week concurrent strength-endurance training intervention significantly improved explosive power and specific performance in elite female triathletes. Key outcomes included notable improvements in squat strength, relative squat strength, and squat jump (SJ) performance, along with significant gains in running and swimming, especially in sprint-distance triathlon. This study confirms the hypothesis that concurrent training enhances both neuromuscular strength and muscle elasticity, ultimately improving sprint-distance performance.

However, the effects on long-distance events, such as the 5000m run and 1500m swim, were less pronounced, highlighting the selective nature of the benefits of concurrent training. The study also suggests the potential for long-term retention of gains made during the intervention, with performance in explosive power and short-distance events sustained even after returning to traditional training.

Future research should focus on optimizing training periodization to maximize the synergies between strength and endurance training, further investigating how concurrent training can be sustained over longer periods to ensure continued benefits. Additionally, validating these findings across diverse athlete populations could provide broader insights into the application of concurrent training in triathlon, leading to more refined, evidence-based training recommendations.

## Supporting information

S1 Fig Actual daily training time. (TIF)

S2 Fig Comparison of weekly average TRIMP between pre-experiment and experiment. (TIF)

S3 Fig Fitness and specialized results between pre-experiment and post-experiment. (TIF)

S4 Fig Fitness and specialized results between pre-experiment and post-experiment (continue). (TIF)

S5 Fig Indicators change at 0, 4 and 8 weeks of follow-up period. (TIF)

S1 Table TRIMP in 8 weeks. (XLSX)

S2 Table Indicator of 12 athletics. (XLSX)

S3 Table Weekly training time. (XLSX)

S4 Table Follow-up indicators. (XLSX)

S1 File Ethical statement. (PDF)

S2 File Ethical statement (translate to English). (PDF)
