# Supplementary figures and images for: Effects of concurrent training on the Chinese female elite triathletes
Source: PLoS One. 2025 Aug 13;20(8):e0329588. doi: 10.1371/journal.pone.0329588 (PMC12349246; doi:10.1371/journal.pone.0329588)

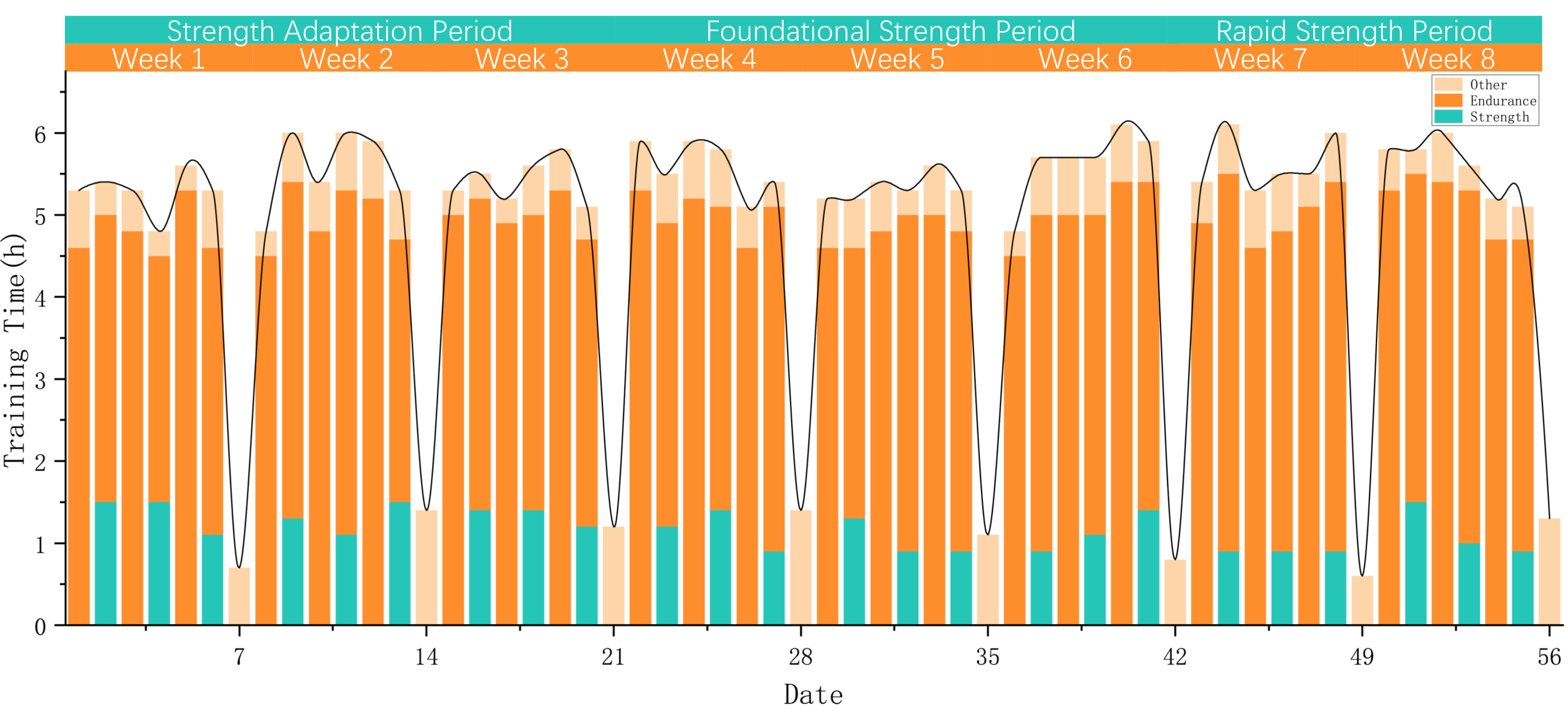

Supplement: S1 Fig — (TIF) [file pone.0329588.s001.tif]

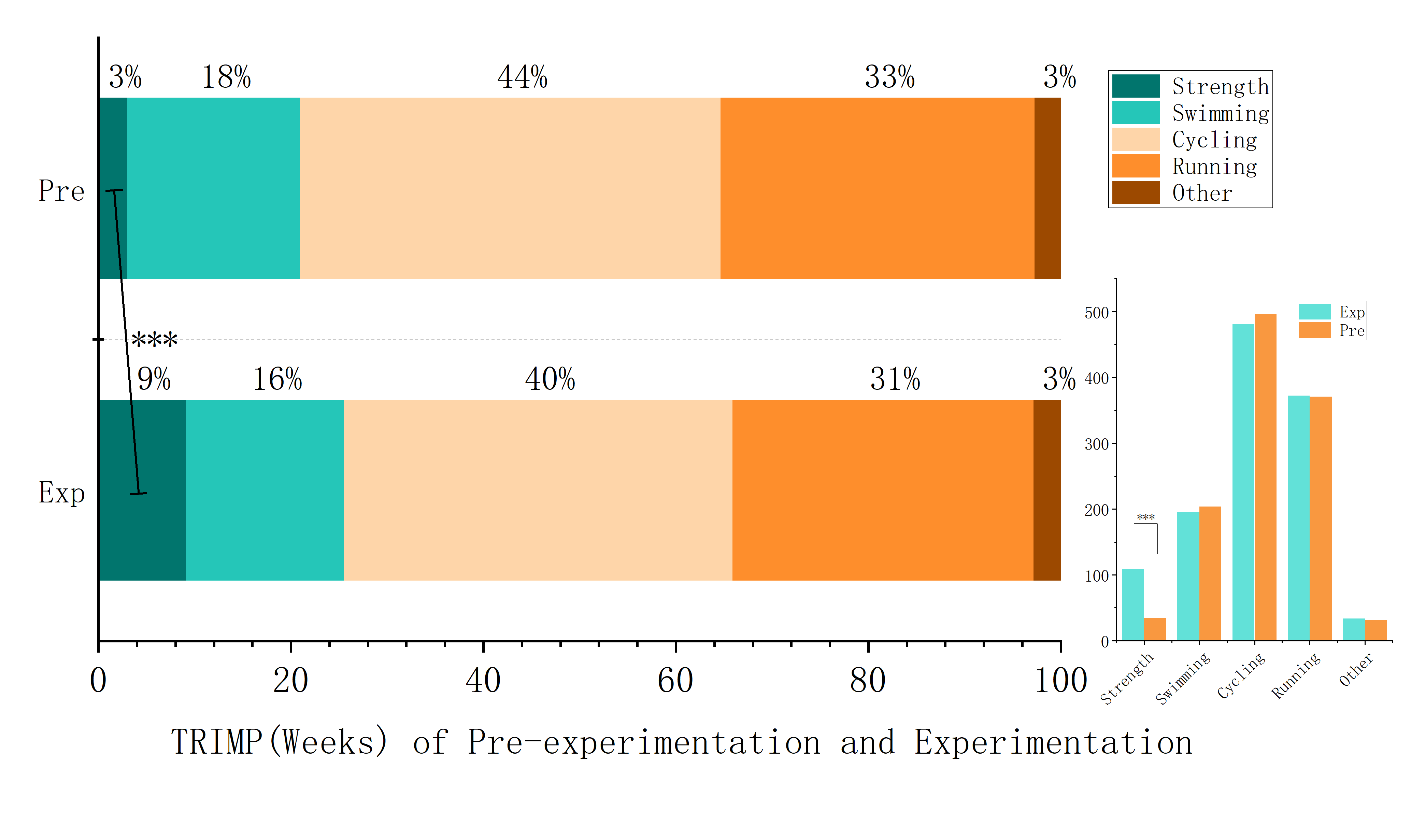

Supplement: S2 Fig — (TIF) [file pone.0329588.s002.tif]

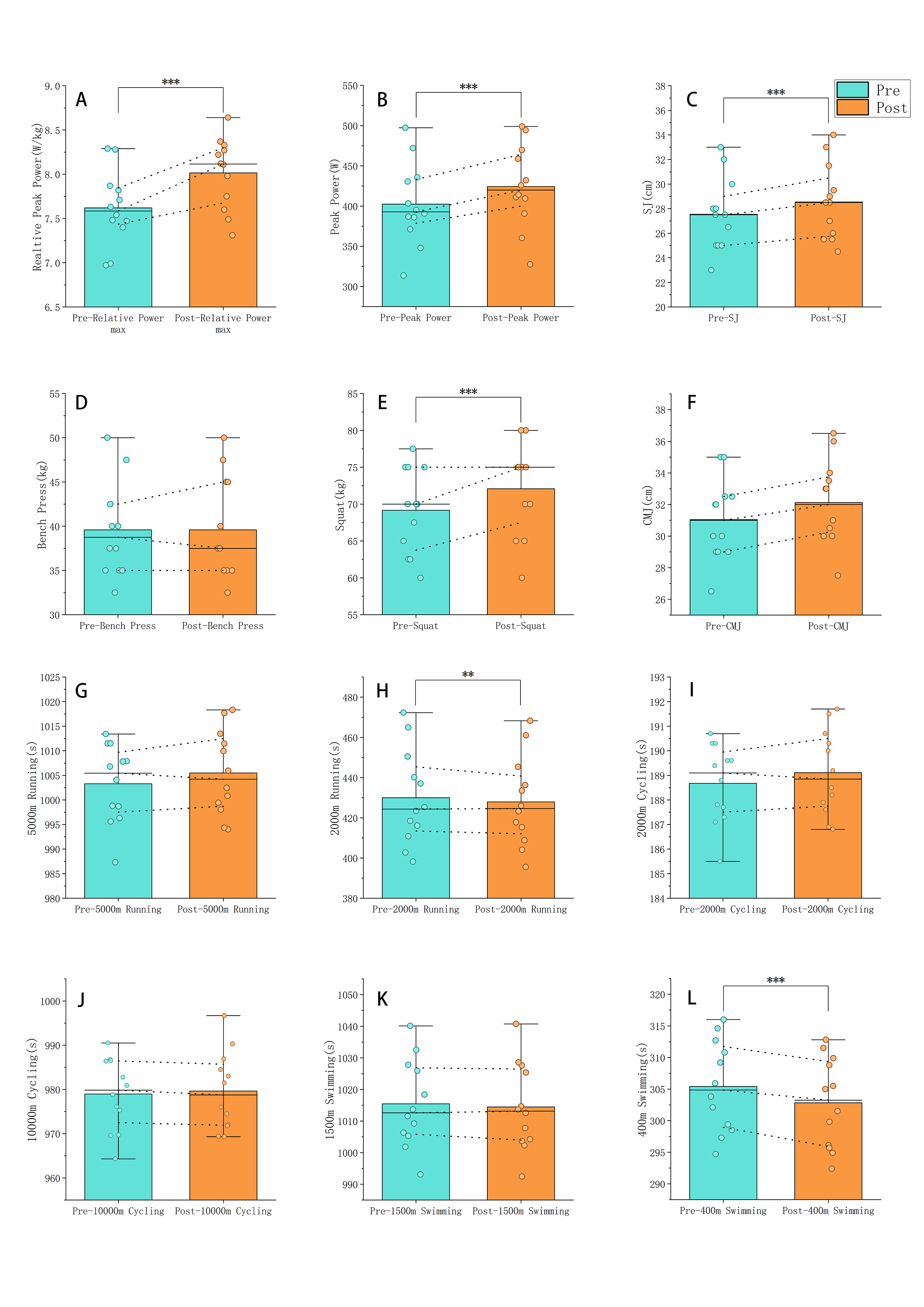

Supplement: S3 Fig — (TIF) [file pone.0329588.s003.tif]

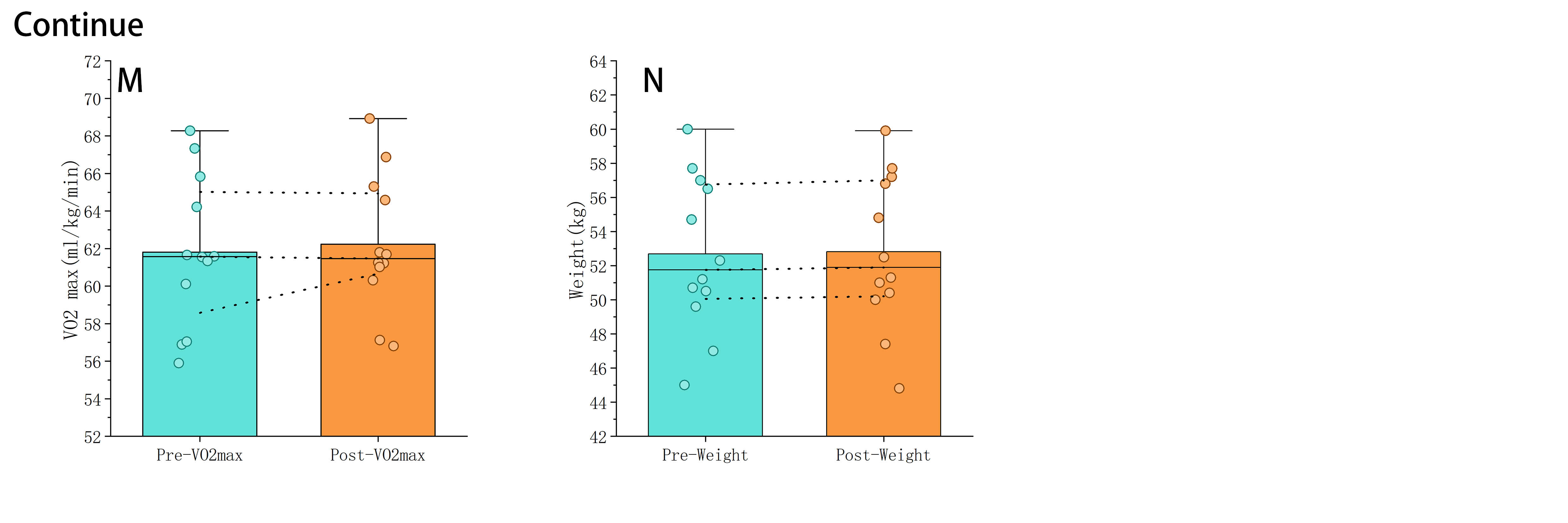

Supplement: S4 Fig — (TIF) [file pone.0329588.s004.tif]

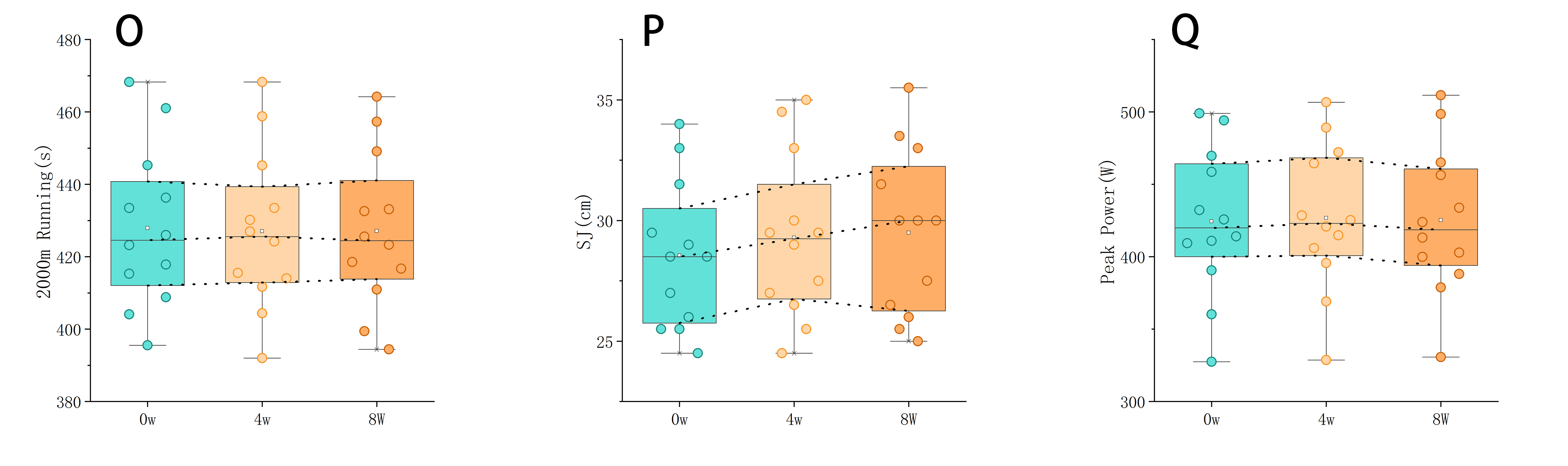

Supplement: S5 Fig — (TIF) [file pone.0329588.s005.tif]
