# Supplementary material for: Effects of concurrent training on the Chinese female elite triathletes
Source: PLoS One. 2025 Aug 13;20(8):e0329588. doi: 10.1371/journal.pone.0329588 (PMC12349246; doi:10.1371/journal.pone.0329588)
Supplement: S1 File — (PDF) [file pone.0329588.s010.pdf]

天津体育学院伦理审查申请表

伦理编号：TJUS 2025-078

项目名称：Effects of Concurrent Training on The Chinese Female Elite Triathletes					
项目负责人	刘承豪	职称	学生	单位/部门	运动训练学院
联系电话	15668116739	课题组成员	刘承豪、张镇宇、夏力、毛睿、靳泽宇、王娇洁、王馨迤、郝诗语、谢云		
申请审查类型	☐申请项目 ☐批准后项目 ☐延续项目 ☐委托项目 ☑发表论文<br>☐其他（请注明）：____				
研究项目经费来源：☐政府 ☐基金会 ☐公司 ☐国际组织 ☑其他					
递交审查资料：☑方案 ☐知情同意书 ☐其他资料：					
是否涉及人体研究：☑是 ☐否			是否涉及动物研究：☐是 ☑否		

研究内容及研究方案摘要：

本研究旨在探讨力量-耐力并行训练对中国国家级女子铁人三项运动员专项表现的影响。共纳入12名符合选拔标准的精英运动员，开展为期8周的训练干预。训练方案采用周期化设计，包含力量适应、基础力量与快速力量三个阶段，同时结合游泳、骑行与跑步等专项耐力训练，训练频率每周6次。研究通过非侵入性方式评估干预前后运动表现变化，包括深蹲1RM、静态/反应跳跃、400米游泳、2000米跑步、短距离铁三模拟赛及最大摄氧量等指标。

为监测训练负荷，本研究采用TRIMP（Training Impulse）方法结合主观评分（sRPE）进行全过程控制。所有测试安排在训练周期前后各一周内完成，并在干预结束后持续进行8周的跟踪测试，以观察干预效应是否可持续。研究过程中不涉及血样、隐私信息或有创检查，所有受试者签署知情同意书。

审查类别： ☑加快审查 ☐会议审查

结论： ☑同意 ☐作必要修改后同意 ☐不同意 ☐暂停或终止试验

申请人（项目负责人）承诺：

以上所填内容均属实，如获批准，我将严格按照提供的方案进行研究，并遵守天津体育学院伦理委员会的相关规定。

申请人（项目负责人）签字：刘承豪

日期：2025 年 6月9 日

伦理委员会审查意见：

经本伦理委员会审查：

1.研究者具备开展该研究的资格。

2.研究方案及知情同意书基本符合伦理要求。

请遵循我国相关法律、法规和规章，并遵循天津体育学院伦理委员会批准的方案和知情同意书开展研究。

天津体育学院伦理委员会（盖章）

批准日期：2025年 6月9 日

备注：此表一式3份，申请人、伦理委员会及科研处各存1份。
