# Supplementary material for: Effects of concurrent training on the Chinese female elite triathletes
Source: PLoS One. 2025 Aug 13;20(8):e0329588. doi: 10.1371/journal.pone.0329588 (PMC12349246; doi:10.1371/journal.pone.0329588)
Supplement: S2 File — (PDF) [file pone.0329588.s011.pdf]

Tianjin University of Sport Ethical Review Application Form

Ethics number：TJUS _____-____

Project Title: Effects of Concurrent Training on The Chinese Female Elite Triathletes					
Project Manager	Chenghao Liu	Title	Student	Department	Department of Exercise
Tel	15668116739	Members	C.L、Z.Z、L.X、R.M、Z.J、J.W、X.W、S.H、Y.X		
Type	□Application Program □Post-approval projects □Continuing projects □Commissioned projects ☑Published Papers □Other (specify)：____________________				
Sources of funding for research projects：□Governments □Foundation □Firms □International organization ☑ Other					
Submission of review information ：☑Programmatic □Informed Consent □Other Information:					
Does it involve human subjects research: ☑Yes □No			Does it involve animal studies: □Yes ☑No		
Summary of the study and research protocol:<br>The aim of this study was to investigate the effects of parallel strength-endurance training on the specialized performance of Chinese national-level female triathletes. A total of 12 elite athletes who met the selection criteria were included in an 8-week training intervention. The training program was designed as a periodized program consisting of three phases of strength adaptation, basic strength and fast strength, combined with specialized endurance training such as swimming, cycling and running, with a training frequency of 6 times per week. The study assessed changes in exercise performance before and after the intervention in a non-invasive manner, including deep squatting 1RM, static/reactive jumping, 400m swimming, 2000m running, short-distance triathlon simulation race and maximal oxygen uptake.<br>To monitor the training load, this study used the TRIMP (Training Impulse) method combined with subjective rating (sRPE) for the whole process control. All tests were scheduled to be completed one week before and after the training cycle, and follow-up tests were continued for 8 weeks after the intervention to see if the intervention effect was sustainable. No blood samples, private information, or invasive tests were involved in the study, and all subjects signed an informed consent form that met the criteria for exemption from ethical review.					
**Review category:** □ Expedited review □ Conference review					
**Conclusion:** □Agree with □Agreed, mutatis mutandis □Disagree □Suspension or termination of the test					
The applicant (project leader) promises:<br>All the contents filled in above are true, and if approved, I will conduct the study in strict accordance with the provided protocol and abide by the relevant regulations of the Ethics Committee of Tianjin Sports Institute.<br>Signature of applicant (project leader).<br>Date:					

Ethics Committee Review Opinion:<br><br>Reviewed by this Ethics Committee:<br>1. The investigators were qualified to conduct the study.<br>2. The study protocol and informed consent form basically meet the ethical requirements.<br>Please follow the relevant laws, rules and regulations of China, and follow the protocol and informed consent approved by the Ethics Committee of Tianjin Sports Institute to conduct the study.<br>**Ethics Committee of Tianjin Sport Institute (seal)**<br><br>Date of Approval:

Remarks: This form is in three copies, one for the applicant, one for the Ethics Committee and one for the Research Office.
